# An Age-Wise Comparison of Human Airway Smooth Muscle Proliferative Capacity

**DOI:** 10.1371/journal.pone.0122446

**Published:** 2015-03-23

**Authors:** Michael Fayon, Annick Andrieux, Imane Bara, Muriel Rebola, André Labbé, Roger Marthan, Patrick Berger

**Affiliations:** 1 Université de Bordeaux, Centre de Recherche Cardio-thoracique de Bordeaux, U1045, F-33000, Bordeaux, France; 2 CHU de Bordeaux, Hôpital Pellegrin-Enfants, Pneumologie Pédiatrique, Centre d’Investigation Clinique (CIC 1401), F-33076, Bordeaux, France; 3 CHU de Clermont-Ferrand, Pediatric Pulmonology and Intensive Care Unit, 63000, Clermont-Ferrand, France; French National Centre for Scientific Research, FRANCE

## Abstract

We compared the proliferation of neonatal and adult airway smooth muscle cells (ASMC) with no/moderate lung disease, in glucose- (energy production by glycolysis) or glucose-free medium (ATP production from mitochondrial oxidative phosphorylations only), in response to 10% fetal calf serum (FCS) and PDGF-AA. In the presence of glucose, cell counts were significantly greater in neonatal vs. adult ASMC. Similarly, neonatal ASMC DNA synthesis in 10% FCS and PDGF-AA, and [Ca^2+^]i responses in the presence of histamine were significantly enhanced vs. adults. In glucose-free medium, cell proliferation was preserved in neonatal cells, unlike in adult cells, with concomitant increased porin (an indicator of mitochondrial activity) protein expression. Compared to adults, stimulated neonatal human ASMC are in a rapid and robust proliferative phase and have the capacity to respond disproportionately under abnormal environmental conditions, through increased mitochondrial biogenesis and altered calcium homeostasis.

## Introduction

Asthma-like symptoms and airway hyper-responsiveness (AHR) are frequently reported in children subsequent to premature birth and/or bronchopulmonary dysplasia [1–3]. At the age of 11 years, children born extremely preterm have significantly more respiratory symptoms than classmates, with twice as many (25 vs. 13%; p < 0.01) having a current diagnosis of “asthma”. Baseline spirometric values are significantly reduced and bronchodilator responsiveness is increased in those born extremely preterm, the changes being more marked in those with prior bronchopulmonary dysplasia [4, 5]. The origin of AHR—genetic factors, lung injury, or abnormal airway development—in these children is unclear [1]. “Asthma” and AHR in preterm neonates are either unrelated or less related to factors commonly known to be implicated in childhood asthma (inheritance, allergy, airway inflammation, and cigarette exposures). These respiratory manifestations thus seem to represent a separate clinical entity [2].

The mechanisms underlying these respiratory manifestations still remain to be investigated but [2], both an arrest in alveolar development and remodeling of airways have already been described. The fibroproliferative or reparative phase of chronic lung disease is histologically characterized by type II pneumocyte hyperplasia, hypertrophy of bronchial and bronchiolar smooth muscle and interstitial and perialveolar fibrosis [6]. Neutrophils play a key role in initiating lung damage by releasing enzymes such as elastase and matrix metalloproteinases that disrupt the lung extracellular matrix [7].

Premature neonates thus have for their postconceptional age normal-sized airways with an increase in amount of bronchial smooth muscle [8]. It is unclear as to whether this increase in airway smooth muscle (ASM) is due to the age-related growth properties of the ASMC (ASM cells), or to the influence of abnormal environmental conditions, or both. It has been shown that ASM mass is particularly increased when subjected to artificial ventilation [8], and hyperoxia has dose-dependent effects on structure and function of developing ASM (enhanced proliferation at <60% oxygen but increased apoptosis at >60%) [9]. It has also been shown that abnormal environmental conditions [10, 11] may easily drive the ASMC into a disease state. Thus, it is important to determine the mechanisms inducing increased airway smooth muscle (ASM) mass and subsequent AHR [12–15], as compared to what is known in asthma.

In adult or infant asthma, a complex network of cytokines and genes expression appear to be deregulated, resulting in inflammation (independent of atopy [16]) and increased production of ASMC. *In vitro*, cultured ASMC from asthmatic patients show growth abnormality [14] i.e. these cells grow more rapidly than those from non-asthmatic patients. Several hypotheses have been put forward to account for this phenomenon such as a decreased expression of the CCAAT/enhancer binding protein alpha (C/EBPalpha), an anti-proliferative transcription factor [17], a decreased expression in sarco/endoplasmic reticulum Ca2+ ATPase (SERCA) [18], an increased expression in transient receptor potential channel (TRPC1), modulating capacitative Ca^2^+ entry in ASMC [19], or different cellular energetic pathways, in particular enhanced calcium-dependent mitochondrial biogenesis [20].

Mitochondrial activation in ASMC plays a major role in both cell proliferation and apoptosis. In a previous study [20], we have shown that mitochondrial mass and oxygen consumption are greater in the ASM from asthmatic adults than those from controls. This feature, which is specific to asthma, was related to an enhanced mitochondrial biogenesis through up-regulation of peroxisome proliferator-activated receptor gamma coactivator (PGC)– 1 alpha, nuclear respiratory factor-1, and mitochondrial transcription factor A. The priming event of such activation is an alteration in ASM calcium homeostasis. Asthmatic ASM is characterized by increased cell growth and proliferation. Thus, ASMC in asthmatic patients is characterized by an altered calcium homeostasis that increases mitochondrial biogenesis, which, in turn, enhances cell proliferation, leading to airway remodeling. This latter finding was very recently confirmed *in vivo*, since the calcium blocker gallopamil was able to decrease ASM mass in a placebo-controlled double-blind trial performed in severe adult asthmatics [21].

The aim of the present study was thus to compare the age-wise proliferation of human isolated cultured ASMC with little or no disease obtained from either neonates or adult patients. We first characterized the human isolated cultured ASMC cells in the context of their regulation of [Ca^2+^]i, cellular proliferation (DNA synthesis and cell counting) and mitochondrial proteins (immunoblotting). Furthermore, we assessed the specific role of mitochondria in ASMC proliferation by comparing ASMC growth in the presence or absence of anaerobic glycolysis. For this purpose, ASMC cell proliferation was determined in both groups of subjects using either glucose or galactose in the culture medium. Indeed, the presence of glucose allows ATP to be produced by both aerobic and anaerobic glycolysis, whereas galactose, only allows cells to produce ATP by mitochondrial oxidative phosphorylation.

## Materials and Methods

### Patient Characteristics

Human lung was obtained at autopsy (< 4 hours after death) or thoracotomy from 5 neonates (Table 1), and 10 adults undergoing lung resection. The neonates were premature and had been ventilated in 4 cases and the last case was post-operative. Smooth muscle cells were taken from the bronchus of Case 4 neonate while others were from trachea. None of the adult donors were asthmatic, and were lifelong nonsmokers or former smokers without COPD. Adult tissue were all of bronchial origin.

**Table 1:** Patients’ characteristics.

Neonates (n°)	Sex	Age at biopsy (d)	Post-Conceptional age at biopsy (d)	Weight (at biopsy) (g)	Cause of death	Lung Disease		
1	F	18	35	3 300	Cerebral anoxia	None		
2	M	2	35	3100	Pulmonary hemorrhage	Mild RDS		
3	M	6	33	2280	Liver failure	None		
4	M	21	43	3650	CLE*	None		
5	M	14	27	1310	Brain hemorrhage	Mild RDS		
**Adults (n°)**	**Sex**	**Age (yrs)**	**FEV1 (%)**	**FEF_25–75_ (%)**	**sGaw (%)**	**TLC (%)**	**RV (%)**	**PaO2 (mmHg)**
1	M	54	89	78	154	100	135	74
2	M	42	106	75	132	126	158	83
3	F	53	99	65	84	109	115	88
4	F	55	113	83	170	89	73	100
5	M	74	80	55	171	103	124	73
6	M	75	108	59	135	92	68	75
7	M	55	109	120	123	99	116	87
8	M	57	91	52	145	123	143	78
9	M	52	113	105	209	113	116	91
10	M	75	88	60	137	94	103	86

*CLE: Congenital lobar emphysema, non-ventilated patient; FEF_25–75_: forced expiratory flow between 25% and 75% of forced vital capacity; FEV1: forced expiratory volume in 1 second; PO_2_: arterial partial pressure of oxygen; RDS: Respiratory Distress Syndrome; RV: residual volume; %: percentage of predicted values; sGaw: specific airway conductance; TLC: total lung capacity.

Collection and use of lung specimens were performed in accordance with the ethics recommendations of the University Hospital of Bordeaux and the Comité de Protection des Personnes of Bordeaux (Law No. 76–1181 of 22 December 1976 on organ harvesting). Biopsy tissues were obtained during surgical procedures or during autopsy, and were fully anonymized before access by the authors. Written informed consent was obtained from parents of pediatric donors and from all living adult donors; consent to medical procedures also included consent to use of discarded tissue for research. The IRB in Bordeaux (CPP = Comité de Protection de Personnes) was informed of the study, but specific approval from the CPP was not required because the research used tissue sampled from autopsy or for other medical care. In France, unless stated otherwise by the parents or next of kin, post-mortem tissues can be used for any scientific studies.

### Isolation and culture of ASMC [22]; (see also S1 Methods)

Segments of trachea or bronchi were dissected and smooth muscle bands were cut, transferred onto culture plates. The cells were incubated at 37°C in 5% CO_2_–95% air and passaged upon confluence. Passages 2–5 only were used for experimentation. The purity of the cells was verified by an indirect immunofluorescence technique. All human ASMC stained positively for smooth muscle actin and myosin.

### DNA synthesis [22]; (see also S1 Methods)

Cells were grown for 24 hours (day-2) in 96-well plates (2000 cells/well), starved for 24 hours in a serum-free DMEM/glucose (ITS medium) (day-1). Cells were then incubated with either growth medium containing10% FCS, or ITS medium in the absence or in the presence of Platelet Derived Growth Factor AA (PDGF-AA) (15 ng/ml) [23], IL-4, IL-6 or Tumour Necrosis Factor (TNFalpha) (all at 100 ng/ml), histamine (10^–^5 M) [24] and SLIGKV (activator of the protease activated receptor PAR2) (10^–^4 M) (day 0). On days 1 to 5, 0.5 mCi *[methy*l-3 H]thymidine was added to each well for an additional 8 h. Cell proliferation was then arrested by placing the culture plates in a -20°C freezer. Cells were harvested on a glass fiber filter and counted on a liquid scintillation analyzer.

### Cell counting [22]; (see also S1 Methods)

Cells were seeded at a density of 25 000 cells in a 75 cm^2^—tissue culture flasks in growth medium (including 10% FCS) for 24 hours to allow for adherence. Cell proliferation was assessed periodically up to 14 days in the presence of growth medium, ITS medium or glucose-free medium. The doubling time of cell growth was obtained from the proliferation curves [25].

### Immunoblotting [22]; (see also S1 Methods)

Proteins were extracted from ASMC incubated in ITS medium for 1 day. Fifteen μg of protein was applied to a 10% polyacrylamide gel and transferred to PVDF membranes which were blocked overnight at 4°C by 5% bovine serum albumin. The immunoblots were then incubated for 2 hours at room temperature with the primary antibodies. A biotinylated goat anti-rabbit antibody was used as secondary antibody. A Streptavidin—Biotinylated Peroxidase kit was used for amplification, and the immunoblots were revealed by chemiluminescence.

### Intracellular calcium signaling [22]; (see also S1 Methods)

ASMC were placed on circular glass coverslips and starved for 1 day in ITS medium as previously described [26]. This medium was supplemented (stimulated cells) or not (control cells) with the pro-inflammatory cytokine TNFalpha (100 ng/ml). Cells were then incubated for 30 min at room temperature in a 2 mM calcium physiological saline solution containing 1 μM of esterified indo-1. A contractile agonist (10^–^3 M histamine) was injected near the cells [24]. The agonist-induced maximal variations in [Ca^2+^]_i_ in each cell was recorded by microspectrofluorimetry [21].

### Statistical analysis

All values are means ± SEM. “n” corresponds to the number of cases. Statistical analysis was performed using the software package, NCSS 6.0.21. Data were analysed for statistical significance using, as appropriate, the unpaired Student *t*-test, the Mann and Whitney U-test or a multivariate analysis of variance (MANOVA). A *p-* value < 0.05 was considered significant.

## Results

### Demographic data

The demographic data of the included neonates and adults are shown in Table 1. Four (Patients 1, 2, 3 and 5) out of 5 neonates were artificially ventilated, and none received systemic or inhaled corticosteroids. The 10 adult non-asthmatic control subjects received no treatment: half of them were lifelong non-smokers whereas the other half were former smokers without COPD.

### Cell proliferation

#### Cell counts (Fig. 1(A))

When incubated with 10% FCS, the maximal number of cells (at day 10) within the 75 cm^2^ culture flasks were 5.69 ± 0.85 x 10^6^ and 2.57 ± 0.36 x 10^5^ regarding neonatal (n = 3 cases) and adult (n = 5 cases) ASMC, respectively (*p* = 0.03). The doubling time of cell growth from neonatal ASMC was 37.0 ± 3.5 hours vs. 52.3 ± 5.3 in adult ASMC (p = 0.037).

**Fig 1:**
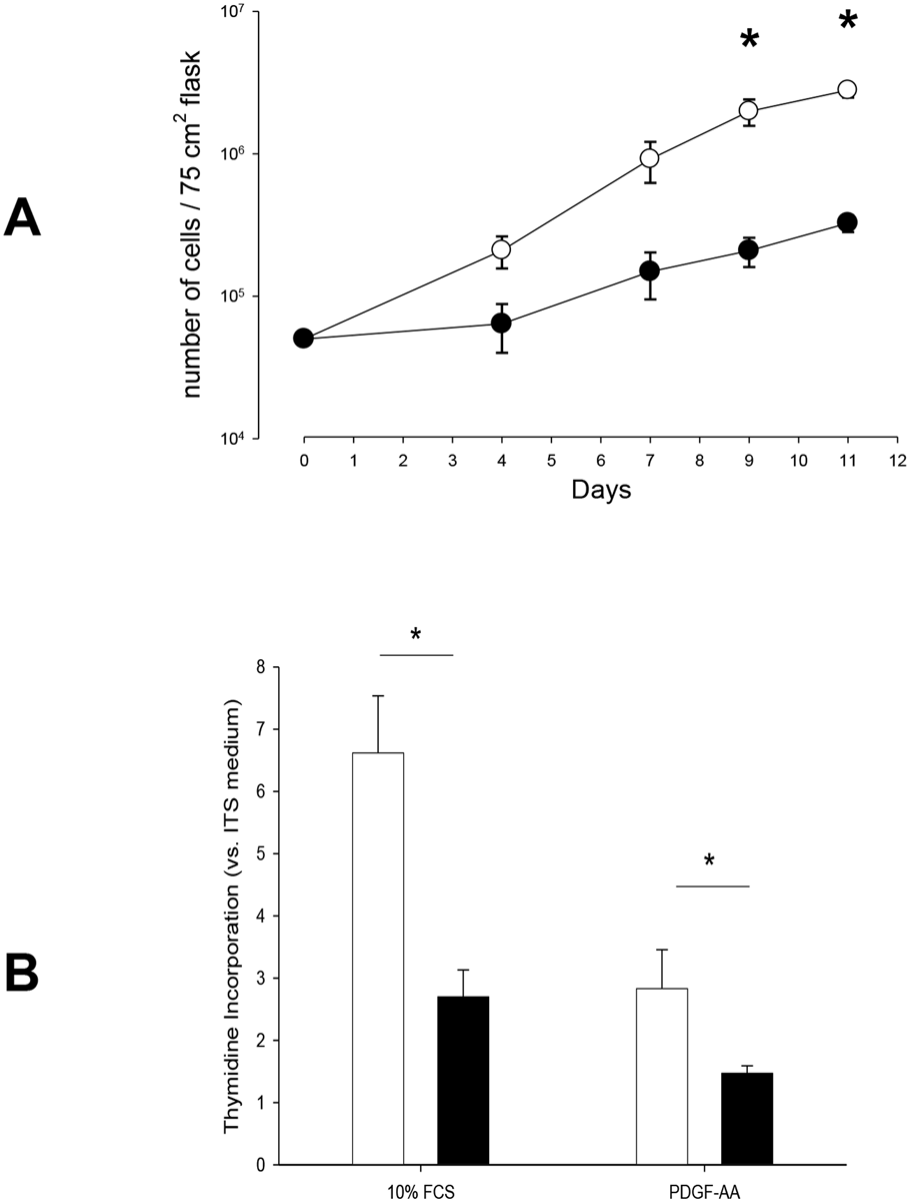
Proliferation of neonatal and adult cultured human airway smooth muscle cells in the presence of FCS and PDGF-AA. **A. Cell counts according to time.** Results are means ± SEM. In glucose + 10% FCS medium, ASMC proliferation was greater in neonatal cells (open symbols, n = 5) vs. adult cells (closed symbols, n = 5). *p < 0.05 neonate vs. adult at the corresponding time using MANOVA. **B. DNA synthesis in adult and neonatal cultured human airway smooth muscle cells.** Results are means ± SEM. Values were normalized to proliferation in ITS medium. Cells cultured in 10% FCS (white bars, neonates, n = 5; black bars, adults, n = 7)), 15 ng/ml PDGF-AA (neonate, n = 4; adults, n = 10). *p < 0.05 using Mann-Whitney U-test.

### DNA synthesis (Fig. 1(B))

When stimulated by 10% FCS, DNA synthesis in neonatal ASMC was significantly enhanced (6.6-fold increase vs. ITS medium) compared to adults (2.7-fold increase) (neonates, n = 5; adults, n = 7; *p* = 0.02). A similar response was observed after stimulation by PDGF-AA (15 ng/ml): 2.6-fold increase in DNA synthesis in neonatal ASMC vs. a 1.5-fold increase in adult ASMC (neonates, n = 4; adults, n = 10; *p* = 0.02).

**Fig 2:**
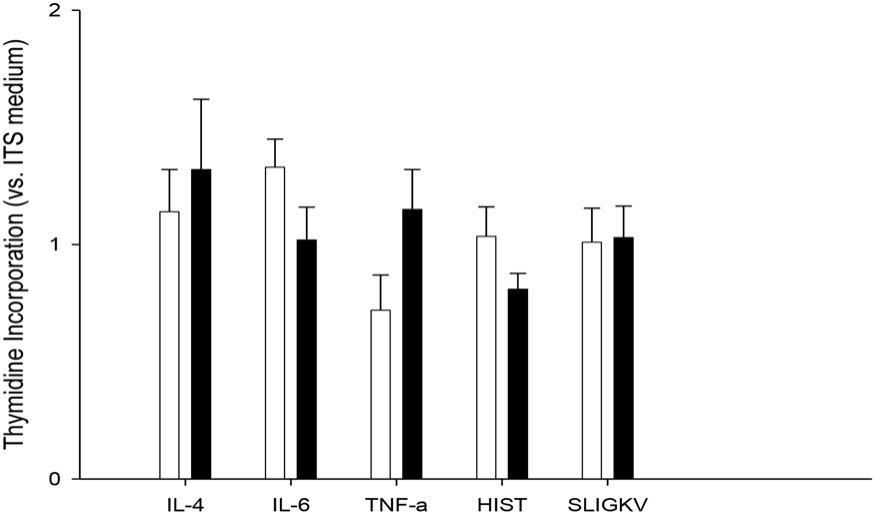
Proliferation of neonatal and adlult cultured human airway smooth muscle cells in the presence of pro-inflammatory stimuli. Results are means ± SEM. Values were normalized to proliferation in ITS medium. Cells cultured in 100 ng/ml IL-4 (black bars, adults; white bars, neonates)), IL-6, TNFalpha or 10^-4^M Histamine, SLIGKV were assayed 24 following synchronization in ITS medium. There were no significant difference between the 2 age groups regarding all experimental conditions (N = 3 to 6 per group, Mann-Whitney U-test).

By contrast, there was no significant difference in DNA synthesis between neonatal and adult ASMC, 24 hours after synchronization of the cell cycles regarding IL-4: 97 ± 23 (n = 3) and 150 ± 31% (n = 4) of baseline values in ITS medium, respectively; IL-6: 128 ± 11% (n = 4) and 120 ± 23% (n = 4), TNFalpha: 73 ± 20 (n = 4) vs. 115 ± 2% (n = 3), Histamine 104 ± 12% (n = 6) and 81 ± 7% (n = 3) or SLIGKV: 101 ± 15 (n = 6) and 103 ± 13% (n = 3) (Fig. 2)

### Analysis of putative mechanisms

#### Mitochondrial mass

**Fig 3:**
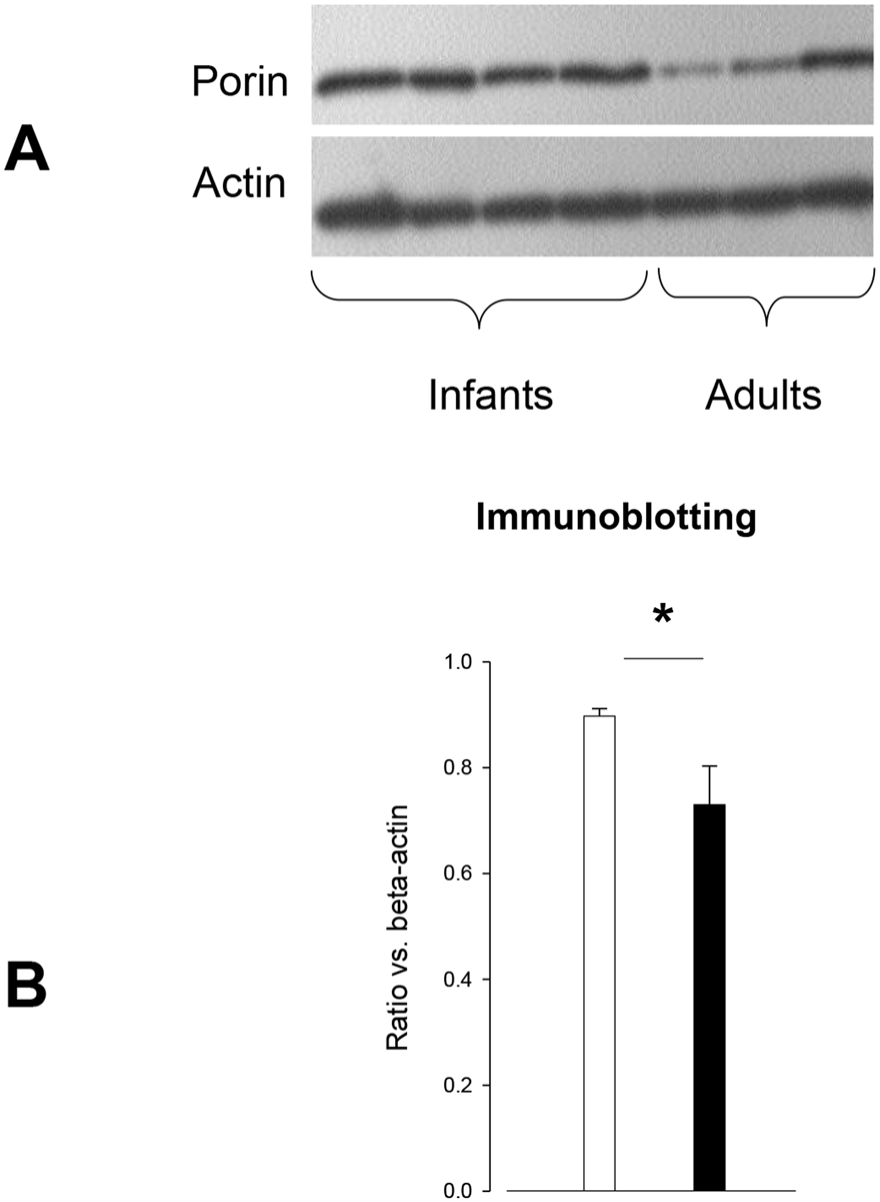
Porin expression according to age groups. **A. Immunoblot for porin and actin in neonatal and adult ASMC. B. Expression of porin in human cultured ASMC.** A significant difference between the two cell populations after incubation in ITS medium for 1 day was found (neonates, white bars, n = 4 and adults, black bars, n = 3, *p < 0.*05* neonate vs. adult using Mann-Whitney U-test.

The mitochondrial mass assessed by the porin level was significantly increased in neonatal ASMC (Fig. 3).

#### Mitochondrial metabolism

**Fig 4:**
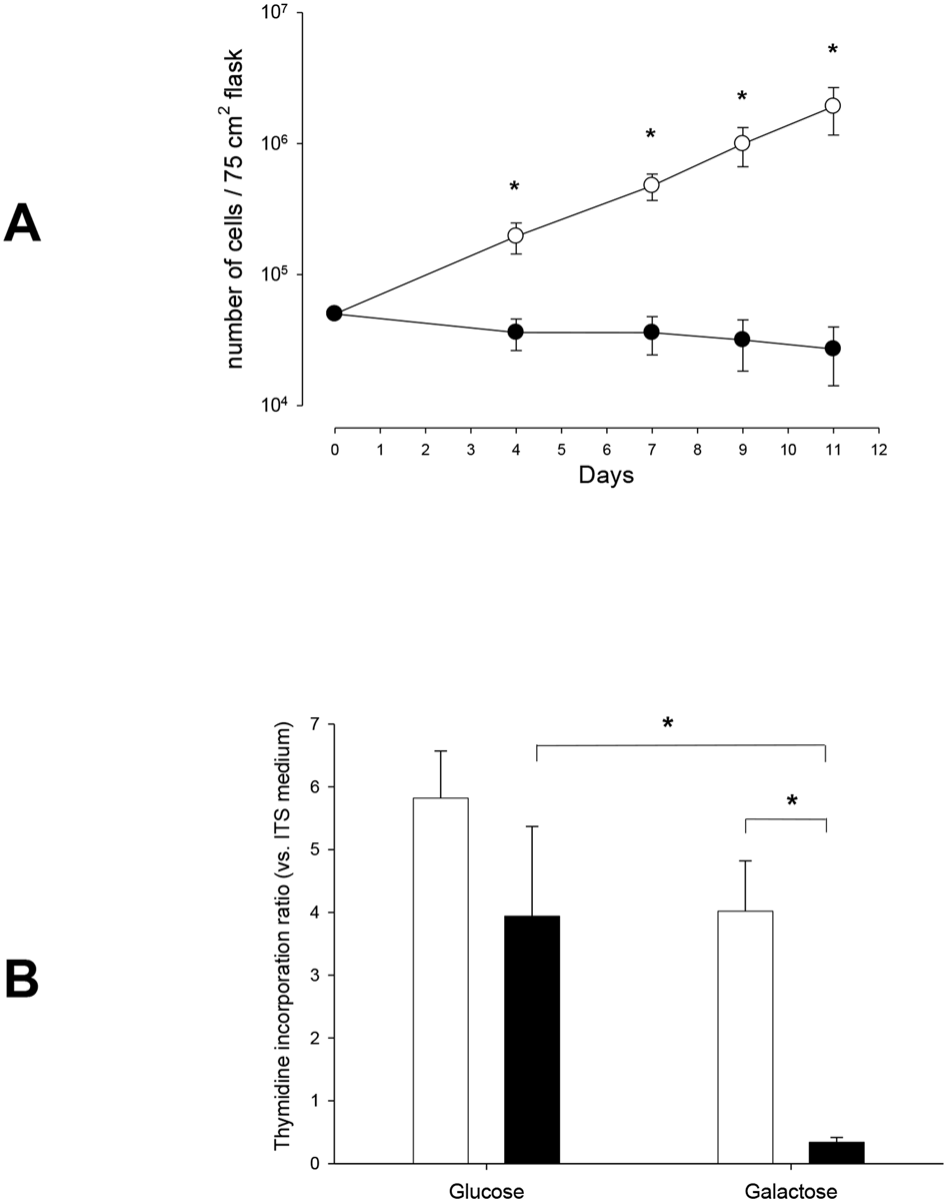
Effect of mitochondrial metabolism (aerobic glycolysis) on ASMC proliferation. **A. Cell count according to the presence of glucose or not (galactose) in the culture medium.** Results are means ± SEM. In galactose + 10% FCS medium, proliferation of neonatal ASMC (open circles, n = 6) is still present, unlike in adult ASMC (closed circles, n = 4). *p < 0.*05* neonate vs. adult at the corresponding time using MANOVA. **B. DNA synthesis according to the presence or absence of glucose in the culture medium.** Values are means ± SEM. After 24h in a glucose-free (galactose) medium, neonatal ASMC (white bars, n = 5), DNA synthesis is still present, in contrast with adult ASMC (black bars, n = 3). *p < 0.05 using Mann-Whitney U-test.

Neonatal ASMC counts increased according to time despite the absence of glucose in the culture medium, which drove cell metabolism to mitochondrial oxidative phosphorylation (Fig. 4(A)). By contrast, under similar experimental conditions, adult ASMC were unable to proliferate and cell counts decreased (Fig. 4(A)). Regarding DNA synthesis, there was a moderate decrease from 5.82 ± 0.75 fold (glucose) to 4.02 ± 0.80 fold (galactose) in neonates (n = 6), whereas in adults (n = 3), there was a major decrease from 3.94 ± 1.43 fold (glucose) to 0.34 ± 0.08 fold (galactose) (p < 0.05) (Fig. 4(B)).

#### Cytosolic calcium response (Table 2)

Since increased mitochondrial mass and metabolism have been related with abnormal calcium homeostasis in adult asthmatic ASMC, we assessed intracellular [Ca^2+^]_i_ responses in both neonatal and adult ASMC [20]. The [Ca^2+^]_i_ response to histamine of neonatal ASMC was increased more than twofold compared to that of adult cells. Moreover, the duration of the calcium peak was also significantly increased in neonatal cells.

**Table 2:** Cytosolic calcium response to histamine (10^-5^M) on day 1 of ITS medium.

[Ca^2+^]i	Neonantal ASMC	Adult ASMC	*p*
**Basal [Ca^2+^]i (nM)**	99.7 ± 15.6	100.0 ± 21.2	NS
**Latency (s)**	13.9 ± 1.7	17.8 ± 5.0	NS
**Relative peak [Ca^2+^]i (nM)**	212.2 ± 27.8	87.6 ± 25.5	0.005
**Duration of peak (s)**	43.3 ± 1.46	38.7 ± 4.7	0.024

Mean ± SEM. N = 6 (Neonate) and 3 (Adult). Comparisons between populations using Mann & Whitney U-test.

#### Others

Neither TRPC1 nor C/EBPalpha expression was altered in neonatal ASMC as compared to that of adult ASMC (see online material).

## Discussion

The present study demonstrates that the proliferative capacity of cultured neonatal ASMC from patients with no or moderate lung disease is enhanced as compared to adult ASMC, indicating the presence of age-wise differences. ASMC in premature infants proliferate fast, and in a robust manner (in a relatively hypoxic or anoxic environment, or with limited energy resources) when stimulated with FCS, which included growth factors and mitogens, as well as PDGF-AA, a relatively weak proliferative stimulus [27]. This finding is consistent with another study in which human fetal ASMC were shown to display robust baseline cell proliferation, compared with adults [9]. This indicates that young age *per se* may lead to this abnormal proliferation, as observed in patient n° 4. The mechanisms implicated share some similarities with adult asthma, i.e. robust ASMC proliferation is associated with increased mitochondrial biogenesis and altered calcium homeostasis.

Enhanced ASMC proliferation may be a normal phenomenon since at this age all the body organs and cells are growing at a faster rate. Bronchial (hyper)-reactivity is crucial for proper in-uterine lung growth. It has been shown that human fetal ASM contacts spontaneously and exhibits pharmacologic responsiveness similar to adult airway smooth muscle [28]. These contractions, possibly exerting effects through phasic changes in intraluminal pressure, may be an important physical force contributing to lung development [28]. Following, birth airway reactivity increases up to the age of 6 years, reaching a plateau after the age of 7 [29]. Early excessive immune responses may induce highly-primed normal neonatal ASMC to proliferate even more and react inappropriately. Although during early development, lung epithelial and mesenchymal growth is regulated by the balance of cytokines and growth factors, prolonged chronic inflammation may drive neonatal ASMC to contribute to the remodeling process [10, 11].

In the present study, neonatal non-asthmatic ASMC were characterized by an increase in mitochondrial mass and the retention of their proliferation property in the absence of glucose. The presence of glucose in the culture medium allows ATP to be produced by both aerobic and anaerobic glycolysis. By contrast, in the absence of glucose, cells can only produce ATP by mitochondrial oxidative phosphorylations. This greater propensity to proliferate of neonatal ASMC was also associated with an enhanced calcium response to histamine as compared to adult cells [20]. Abnormal environmental conditions [10, 11] may easily drive the ASMC into a disease state. When comparing adult asthmatic ASMC proliferation to that of normal adult ASMC [12–14, 20, 30], we found that mitochondria play a key role in the increased proliferation of these asthmatic ASMC, through increased mitochondrial biogenesis and an altered calcium homeostasis [20]. In contrast to the present study, Hartman et al. have shown that although fetal ASM cells show robust [Ca^2+^]i responses to bronchoconstrictor agonists, these responses are generally smaller and slower than in adult ASM cells, suggesting relative immaturity of the regulatory apparatus [9].

The striking similarities between neonatal ASMC and adult asthmatic ASMC (robust ASMC proliferation, increased mitochondrial biogenesis, altered calcium homeostasis) may suggest that the latter cells may not follow the usual age-wise decrease/stabilisation in airway reactivity [31]. Of note, some characteristics of neonatal ASMC proliferation are different from those of asthmatic adults. The proliferative effect of PDGF was clearly increased in neonatal ASMC compared to adult non-asthmatic ASMC whereas it was similar in adult asthmatic or non-asthmatic ASMC [32]. Similarly, asthmatic adult ASMC proliferation appeared to be related to decreased expression of the antiproliferative transcription factor (C/EBPalpha) [17] and/or increased expression of TRPC1 [19], whereas expression of both protein was similar in neonatal ASMC and adult non asthmatic ASMC. Increased proliferation may be due to decreased apoptosis, which was not specifically assessed in the present study. However, the presence of decreased apoptosis in adult ASMC was unlikely since in a study conducted in our own laboratory, apoptosis was similar in asthmatic, non-smokers and smokers, despite increased ASMC proliferation in asthmatic vs. control subjects [32].

The present study presents some limitations. First, the adult tissue was all bronchial derived (6–8^th^ generation) while the neonatal tissue was predominantly tracheal-derived (4 of 5 specimens). Significant differences in ASMC properties have been reported depending on the derivation—tracheal vs. bronchial and then the level of bronchial. It has been shown that asthma is a disease of the entire respiratory tract [33], and BALB/c mice demonstrate an allergen-induced increase in smooth muscle content throughout all generations of airways [34]. Compared to large airways, numerous studies have demonstrated that similar but more severe inflammatory and structural changes occur in the distal lung and lung parenchyma [33]. In the present study, since DNA replication was more pronounced in the proximal airways (in infants), it is likely that similar or a greater degree of replication would have been observed more distally in infants. Second, the cause of death may have been a confounder in neonates, despite the fact that we included neonates whose cause of death was rapid, and lung disease inexistent or mild. Third, the subjects were predominantly male, although the sex ratio was similar in the neonatal and adult populations. Finally, the extent of airway remodeling on lung biopsies was not determined in the present study.

## Conclusion

We have demonstrated age-wise differences in human ASMC capacity. The enhanced proliferation response of neonatal ASMC with moderate lung disease to stimulation by growth factors may be an intrinsic characteristic of neonatal ASMC. These highly-primed normal neonatal ASMC may proliferate even more and react inappropriately in abnormal environmental conditions, unlike adult ASMC.

## Supporting Information

S1 Fig DNA replication of adult and neonatal cultured human airway smooth muscle cells according to percent fetal calf serum in culture medium (A) and culture time in 10% fetal calf serum medium B). Results are means ± SEM. (A) Fold increase in Thymidine incorporation (vs. ITS medium) in adults (n = 3, closed circles) and neonates (n = 3, open triangles). (B) Each symbol corresponds to an individual patient for the respective time point. Values are absolute counts per minute. Cells cultured in 10% FCS (adults, n = 3 (closed circles), neonates, n = 3 (open circles)) were assayed daily for 7 days following synchronization in ITS medium.
(TIF)

S2 Fig Expression for C/EBPalpha, TRPC-1 and PGC1α in human cultured neonatal (white bars, n = 4) and adult (black bars, n = 3) ASMC. No significant difference between the two cell populations after incubation in ITS medium for 1 day was found. Below are representative blots for C/EBPalpha.
(TIF)

S1 Methods Methods and Preliminary data. (DOC)
