# Supplementary figures and images for: An Age-Wise Comparison of Human Airway Smooth Muscle Proliferative Capacity
Source: PLoS One. 2015 Mar 23;10(3):e0122446. doi: 10.1371/journal.pone.0122446 (PMC4370680; doi:10.1371/journal.pone.0122446)

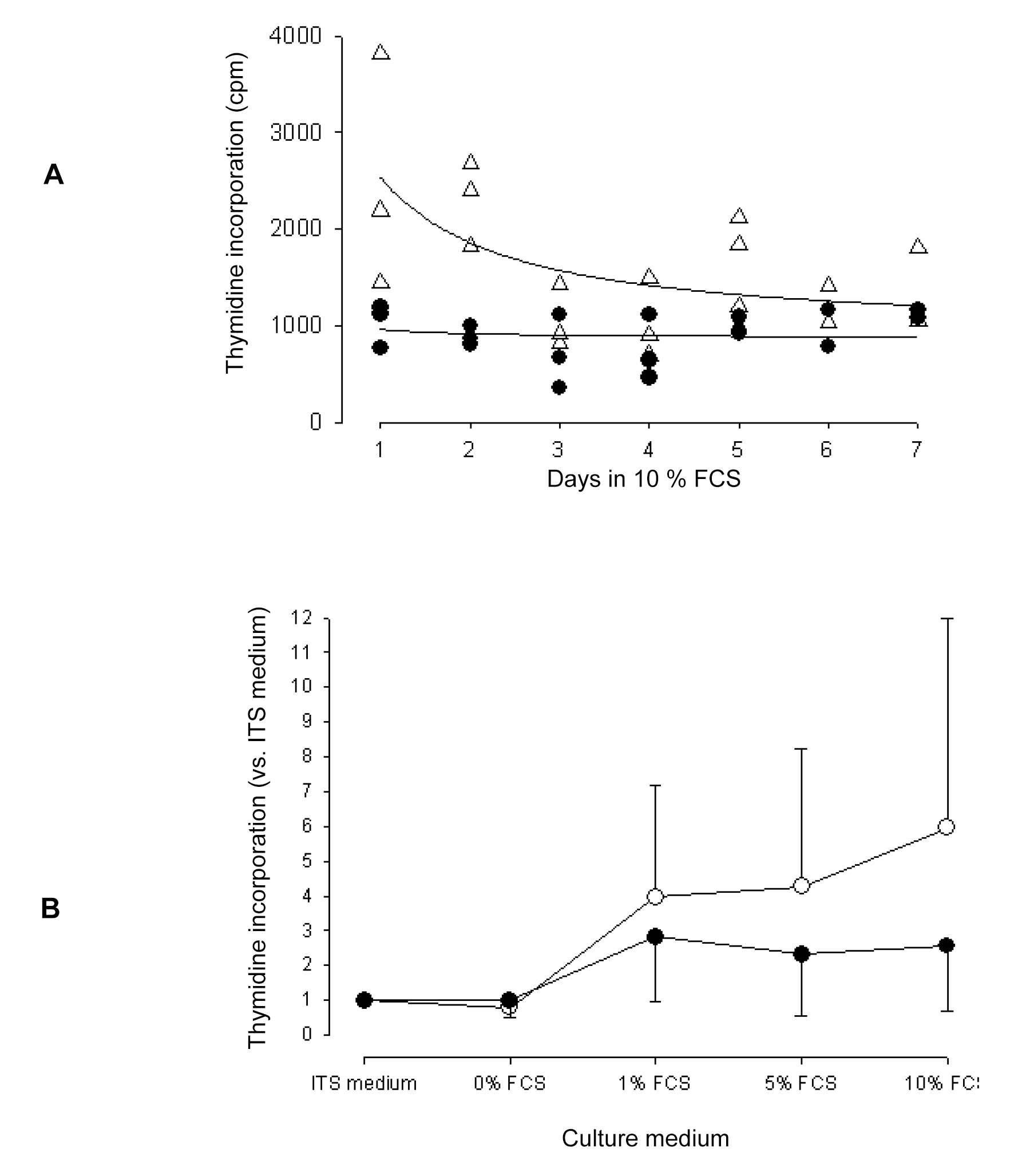

Supplement: S1 Fig — Results are means ± SEM. (A) Fold increase in Thymidine incorporation (vs. ITS medium) in adults (n = 3, closed circles) and neonates (n = 3, open triangles). (B) Each symbol corresponds to an individual patient for the respective time point. Values are absolute counts per minute. Cells cultured in 10% FCS (adults, n = 3 (closed circles), neonates, n = 3 (open circles)) were assayed daily for 7 days following synchronization in ITS medium. (TIF) [file pone.0122446.s001.tif]

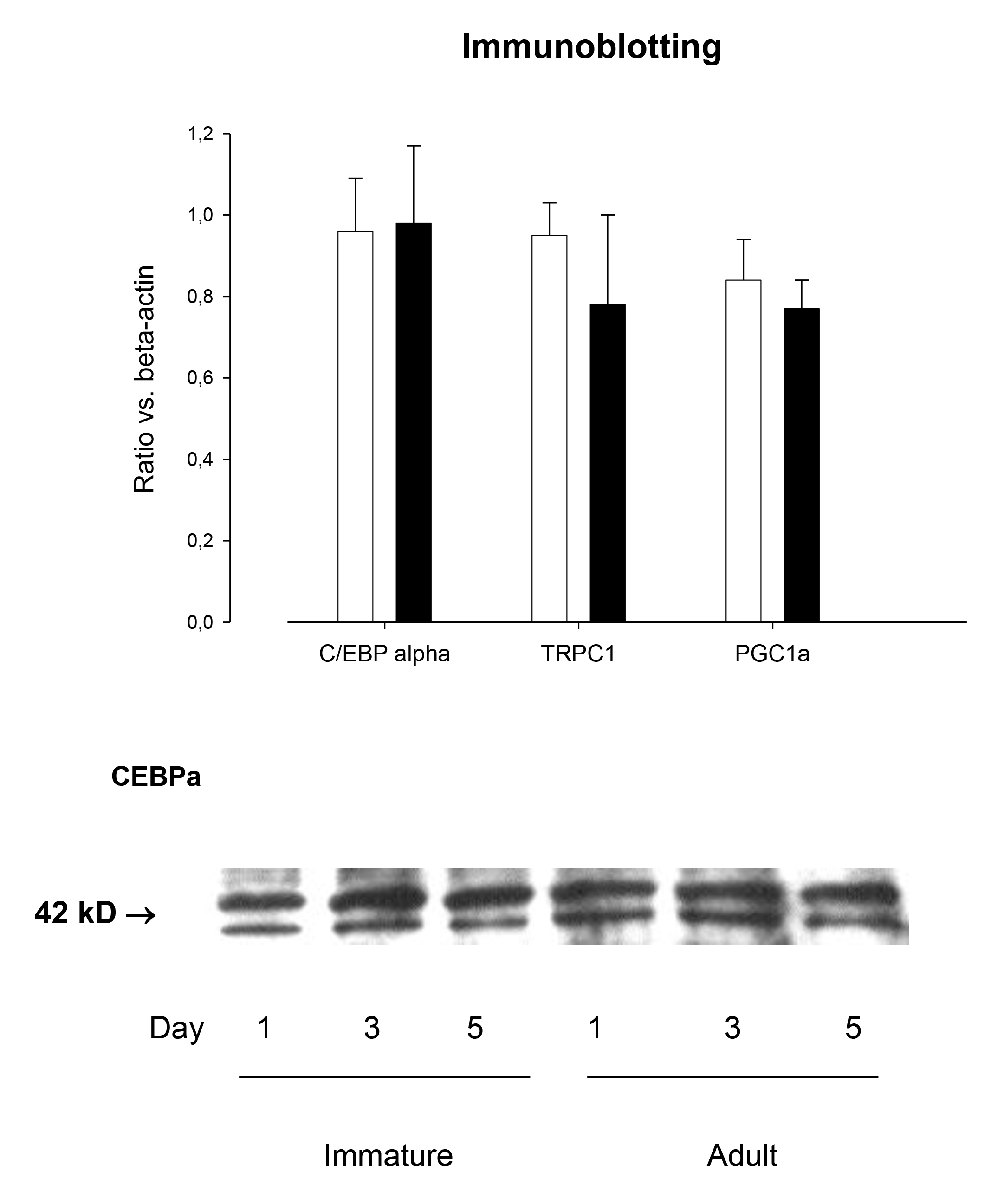

Supplement: S2 Fig — No significant difference between the two cell populations after incubation in ITS medium for 1 day was found. Below are representative blots for C/EBPalpha. (TIF) [file pone.0122446.s002.tif]
