# Supplementary material for: An Age-Wise Comparison of Human Airway Smooth Muscle Proliferative Capacity
Source: PLoS One. 2015 Mar 23;10(3):e0122446. doi: 10.1371/journal.pone.0122446 (PMC4370680; doi:10.1371/journal.pone.0122446)
Supplement: S1 Methods — (DOC) [file pone.0122446.s003.doc]

***Supplemental Information***

**Methods and Initial Data**

**Isolation and culture of human neonatal and adult ASMC.**

The tracheae and bronchi were washed three times with sterile DMEM/glucose (Invitrogen, Cergy Pontoise, France) containing 100 U/ml penicillin, 100 µg/ml streptomycin, and 0.25 µg/ml amphotericin B (Invitrogen). Segments of trachea (babies) or bronchi (adults) were dissected free from the surrounding parenchyma in the same medium at room temperature. The epithelium was removed to expose the underlying bands of smooth muscle, which were then gently separated from the underlying connective tissue in small bundles. The smooth muscle bands were cut into squares measuring 1-2 mm^2^ and transferred onto six-well culture plates and covered with a minimal amount of growth medium containing DMEM/glucose, 10% (vol/vol) FCS (Invitrogen), 2 mM L-glutamine (Invitrogen), 1 mM sodium pyruvate (Sigma Chemical, Saint Quentin Fallavier, France), 1% (vol/vol) nonessential amino acid mixture (Sigma Chemical), 100 U/ml penicillin, 100 µg/ml streptomycin, and 0.25 µg/ml amphotericin B (Invitrogen). The cells were incubated at 37°C in a humidified atmosphere of 5% CO_2_-95% air. The medium was replenished every 3 days. Cells were passaged once they reached confluence (after 6 to 8 weeks), and passages 2-5 were used for experimentation. Cells were rinsed twice with HBSS and then passaged with trypsin-EDTA (both from Invitrogen).

**Cell characterization by immunocytochemistry.**

To assess the purity of the cells, an immunocytochemical method was employed using an indirect immunofluorescence technique [1]. Briefly, after fixation of ASMC in methanol, anti-αactine (clone 1A4, Sigma), anti-myosin (clone hSM-V, Sigma), anti-epithelial cell cytokeratin (Sigma), antiendothelial cell factor VIII (Dako), and anti-fibroblast cellular surface protein (clone 1B10 Sigma) primary antibodies were used to confirm that the cells were ASMC. All human ASMCs stained positively for smooth muscle actin and myosin. An irrelevant antibody served as a control.

### DNA synthesis

Once harvested by trypsinization, cells were grown in 96-well plates (2000 cells/well), starved for 24 hours in a serum-free DMEM/glucose (ITS medium) supplemented with 10 µg/ml insulin, 5.5 µg/ml transferrin, 5 ng/ml selenium, 0.5 µg/ml BSA, 4.7 µg/ml linoleic and oleic acid (Sigma Chemical), 2 mM L-glutamine (Invitrogen), 1 mM sodium pyruvate (Sigma Chemical), 1% (vol/vol) nonessential amino acid mixture (Sigma Chemical), 100 U/ml penicillin, 100 µg/ml streptomycin, and 0.25 µg/ml amphotericin B (Invitrogen). Cells were then incubated for up to 7 days with either growth medium containing up to 10 % FCS, or ITS medium in the absence or in the presence of PDGF-AA (15 ng/ml), IL-4, IL-6 or TNFalpha (all at 100 ng/ml from R&D systems, Minneapolis, USA), histamine and SLIGKV (an oligonucleotidic sequence that activates the tryptase PAR2 receptor) (both at 10^-4^ M). After the incubation period, 0.5 mCi *[methy*l-3 H]thymidine (Amersham, Buckinghamshire, U.K.) was added to each well for an additional 8 h. Cell proliferation was then arrested by placing the culture plates in a -20°C freezer. Cells were harvested on a 0.7-mm-pore glass fiber filter and counted on a 2000CA TriCarb liquid scintillation analyzer [2].

**Cell counting**

Cells were seeded at a density of 25 000 cells in a 75 cm^2^ - tissue culture flasks (Beckton-Dickinson Biosciences, Le Pont de Claix, France) in growth medium (including 10 % FCS, see above) for 24 hours to allow adherence. Cell proliferation was assessed periodically up to 14 days in the presence of growth medium, ITS medium or glucose-free medium (i.e. same growth medium as above without glucose, substituted with galactose). Briefly, cells were washed twice with HBSS solution (Invitrogen) and then treated with trypsin and EDTA (0.05 percent and 0.53 mM, respectively) for 5 minutes. The flask was then agitated until all the cells were unstuck, as evidenced by manual vision under a microscope. After centrifugation (5000 rpm, 5 mn, 4°C), ASMC were counted in duplicate for each flask with the use of a Neubauer improved hemocytometer.

**Immunoblotting**

Proteins were extracted from non-proliferating ASMC incubated in ITS medium for 1 day as previously described [3]. They were fractionated according to size, as follows: 15 µg of total protein was applied to a 10% polyacrylamide-sodium dodecyl sulfate gel and transferred to Immobilon^TM^-P PVDF membranes (Millipore, MA, USA). The membranes were blocked overnight at 4°C by the addition of 5 % bovine serum albumine. The immunoblots were then incubated for 2 hours at room temperature with a primary polyclonal rabbit anti-C/EBPalpha antibody (Santa Cruz Biotechnology, Heidelberg, Germany), TRPC1(ACC-010, Alamone laboratories,) mtTFa, PGC1-alpha (SC-28200 and SC-13067, respectively, Santa Cruz Laboratories) and Porin (MSA03, MitoSciences). A biotinylated goat anti-rabbit antibody (Dako, Trappes, France) was used as secondary antibody. A Streptavidin - Biotinylated Horseradish Peroxidase kit (Dako) was used for amplification, and the immunoblots were revealed by enhanced chemiluminescence (Uptima, Interchim, Montluçon, France). For quantification we used ImageJ 1.43u software (USA)*.*

### Intracellular calcium signaling

Once harvested by trypsinization, cells were placed on 14-mm circular glass coverslips and starved for 1 day in ITS medium. This medium was supplemented (stimulated cells) or not (control cells) with the pro-inflammatory cytokine TNFalphaα (100 ng/ml). Cells were then rinsed and incubated for 30 min at room temperature in a 2 mM calcium physiological saline solution containing 1 µM of esterified indo-1 (indo-1 AM) as described previously [1]. A contractile agonist (histamine) at a concentration of 10^-3^ M, was micro-injected in the immediate vicinity of the cells. The agonist-induced maximal variations in [Ca^2+^]_i_ in each cell was recorded by microspectrofluorimetry using an epifluorescent microscope (Diaphot 300, Nikon) according to a previously described technique [1].

**Results**

**DNA synthesis**

***According to the culture medium***

In a preliminary study, neonatal and adult ASMC cells were cultured for 24 hrs in ITS medium or medium enriched with 1 to 10 % FCS. Maximal [methyl-3 H]thymidine incorporation was observed in the presence of 10 % FCS, in particular regarding neonatal ASMC (S1 Fig). This concentration of FCS was thus used in subsequent studies.

***According to time***

Cultured cells assayed 1 and 5 days in ITS medium showed similar levels of incorporation of *[methy*l-3 H]thymidine : day 1, 440.9 + 147.4 vs. 454.1 + 49.9; day 5, 449.1 + 89.4 vs. 387.6 + 81.0 in neonatal (n = 5) and adult (n = 6) ASMC, respectively (NS).

Cultured cells assayed up to 7 days in 10 % FCS medium showed enhanced incorporation of *[methy*l-3 H]thymidine in neonatal cells compared to adults, in particular for short culture times (days 1 and 2) (S1 Fig). We thus chose a single 24-hour time point for subsequent studies in which cells were stimulated by growth factors and inflammatory mediators.

***Study of possible intrinsic mechanisms*** (S2 Fig)

The expression of the anti-proliferative transcription factor C/EBPalpha protein, the Ca^2+^-permeable channel TRPC-1 and the master activator of mitochondrial biogenesis PGC1α in neonatal and adult cultured ASMC was similar.

**Figure Legends**

**S1 Fig. DNA replication of adult and neonatal cultured human airway smooth muscle cells according to percent fetal calf serum in culture medium (A) and culture time in 10 % fetal calf serum medium B).** Results are means + SEM. (A) Fold increase in Thymidine incorporation (vs. ITS medium) in adults (n = 3, closed circles) and neonates (n = 3, open triangles). (B) Each symbol corresponds to an individual patient for the respective time point. Values are absolute counts per minute. Cells cultured in 10 % FCS (adults, n = 3 (closed circles), neonates, n = 3 (open circles)) were assayed daily for 7 days following synchronization in ITS medium.

**S2 Fig. Expression for C/EBPalpha, TRPC-1 and PGC1α in human cultured neonatal (white bars, n = 4) and adult (black bars, n = 3) ASMC.** No significant difference between the two cell populations after incubation in ITS medium for 1 day was found.Below are representative blots for CEBPalpha.

**Supplementary references**

1. Fayon M, Rebola M, Berger P, Daburon S, Ousova O, Lavrand F, et al. Increased secretion of leukemia inhibitory factor by immature airway smooth muscle cells enhances intracellular signaling and airway contractility. Am J Physiol Lung Cell Mol Physiol. 2006;291:L244-51. PubMed PMID: 16489116.

2. Berger P, Girodet PO, Begueret H, Ousova O, Perng DW, Marthan R, et al. Tryptase-stimulated human airway smooth muscle cells induce cytokine synthesis and mast cell chemotaxis. Faseb J. 2003;17:2139-41. PubMed PMID: 14500550.

3. Fayon M, Dumas De La Roque E, Berger P, Begueret H, Ousova O, Molimard M, et al. Increased relaxation of immature airways to {beta}2-adrenoceptor agonists is related to attenuated expression of postjunctional smooth muscle muscarinic M2 receptors. J Appl Physiol. 2004;98:1526-33. PubMed PMID: 15579574.
